# The Valuation of Gifts in Kind

**Published:** 1915-08-14

**Authors:** 


### KING EDWARD'S HOSPITAL FUND FOR LONDON STATISTICS.*

### The Valuation of Gifts in Kind.

It will be remembered that in the cases of several general hospitals in London in-patients are required to provide for their own use certain necessaries, such as tea, butter, and sugar, and that it was shown in one of these articles that, apart from the unwisdom of allowing as part of the hospital diet regular supplies of food of anonymous origin, a difficulty arose in respect of the accounts. The editor of the statistical return duly marks this point by a note in Section V. of the Report. The confusion arising through this unusual feature will apply to the accounts for 1914, but not to those for 1915, for, under a recent ruling of the Central Funds, certain gifts in kind must be valued, counted as income, and also appear upon the expenditure side to adjust the figures for the department to which they belong. This ruling is a long stride in the direction of completeness.

### Linen, Coal, Brandy, Beef.

For the guidance of the Metropolitan hospitals a circular has been issued indicating the nature of gifts that are to be so valued. They are articles purchased through allied associations and food or things in general use for patients and nurses, etc. The circular points out that some hospitals have outside organisations such as "Linen Leagues," which collect money and buy linen for the hospital; but because this supply has been a "gift" it has not been entered in the accounts. Again, some hospitals have valuable gifts of coal, brandy, and (particularly this year) generous presents of beef, mutton, butter, and other foodstuffs, through the Chamber of Commerce and Colonial Governments.

It is obvious that the value of all these things should be appropriately included in the accounts; if this is not done the comparison with institutions not so fortunate would be an unfair one. To those who accept the theory that cost of washing and cost of renewal of linen should, as suggested in the last article on these statistics, be always considered side by side, the published results of the efforts of Linen Leagues will be peculiarly interesting.

It is stated that the new rule is not meant to apply to anything which is not in general use for patients or nurses, such as gifts of game or fruit. Strictly speaking, these luxuries help to decrease expenditure, but how much is thereby saved it might be difficult to estimate. Therefore, the three Funds consider that gifts of things not in general use may be looked upon as "extras," and need not be entered in the accounts.

### Gift Books and the Annual Report.

Probably in many hospitals already a "Gifts Book" is kept, because a far-seeing secretary understands the importance of duly marking the appreciation of the management for those gifts in kind that friendly people make from time to time to cheer the patients. It is an excellent policy to print a list of these presents in the annual report, and to be careful to send a copy to each person mentioned. These gifts, even when they take the humble form of a few flowers or some illustrated periodicals, are of more importance, sometimes, than their equivalent in cash, because they represent, in many instances, a marked amount of personal trouble, sympathy, and interest. Many a substantial supporter of a hospital has commenced his connection with the charity by making some simple gift, and stories could be told of legacies that are the outcome of circumstances of similar origin.

Every institution which is under the wing of the Funds must now keep a Gifts Book. For purposes of posting, certain gifts, of the nature indicated above, will be entered with a note of the estimated monetary value; we venture to suggest, however, that every gift in kind, however small, be recorded therein, whether the valuation has to be extended or not, that the list shall be concisely printed in the annual report (without values, of course), and a copy of the publication sent to each donor.

### Contract and Comparative Prices.

Section IV. of the Statistical Report deals with the prices for certain articles of food, drugs, and household requisites returned as being paid at the close of the previous year by the 106 hospitals dealt with in the Report. As the prices are, at the publication of the statistics, already eight months old, and as they may, and probably often do, represent contract prices of quite as long a period prior to the date quoted, the figures are more on the interesting than the useful side. It should, however, be said that the information contained in the table has, in a much more elaborate form, been supplied to the constituent hospitals well in advance of the Blue-book. The separate publication now circulated privately was at one time issued to the Press, and has in these columns received extended notice; in these circumstances we do not attempt to criticise the merits of the figures now published.

### A Mistaken View.

It has been said that the publication of comparative prices and of methods of carrying through the more important of the many operations that come under the management of the hospital secretary would expose the efficient officer to constant worrying inquiries from those who are more indolent and less inventive or painstaking. We do not agree. In the profession of medicine there are no discoveries and no perfected methods of treatment that are not immediately presented to the observation of those entitled to participate in the knowledge of them. Copyright or patent in these things, intended for the benefit of mankind, are not dreamed of. The earnest hospital officer will in the same spirit cheerfully communicate any information that will be of service to his fellow-workers.

* Previous articles appeared on October 10 and December 12, 1914, and January 23, February 6 and 20, March 20, April 3, May 15, and June 26, 1915.

The Present Importance of Comparisons.

To return in detail to the practical matters of everyday institutional life. In view of the dearness of supplies of all kinds, and the difficulty in obtaining the services of helpers in all grades, the comparison of conditions prevailing at the individual hospitals is at the present time of exceptional value. It is necessary, however, to obtain recent information on these points, as the conditions change materially in a very short space of time. We are now in the position of having to seek out the sellers instead of having them come to us, and instead of there being a waiting list of applicants for employment there are usually vacant appointments in every department.

Information in respect of the methods in use at different hospitals of carrying out work which is sometimes performed by outside agencies and sometimes by permanent officers or servants, would just now be useful as well as interesting. On the scientific side, for instance, we may mention the *x*-ray work and the examination of blood specimens. In the nursing departments there are the conditions of service and rates of pay of the sisters and nurses. Under general housekeeping expenditure information as to porters' wages, and in respect of window-cleaning, chimney-sweeping, and floor-polishing, whether these are put out now or in normal times, and how often the work is carried out.

These few points occur to us at the moment of writing, but there must be others upon which collected information would, without doubt, be of service to the hospital administrator. We should, therefore, welcome the experience of our readers on these points.

